# Hypertension in Acromegaly in Relationship to Biochemical Control and Mortality: Global ACROSTUDY Outcomes

**DOI:** 10.3389/fendo.2020.577173

**Published:** 2020-11-30

**Authors:** Greisa Vila, Anton Luger, Aart Jan van der Lely, Sebastian J. C. M. M. Neggers, Susan M. Webb, Beverly M. K. Biller, Srinivas Valluri, Judith Hey-Hadavi

**Affiliations:** ^1^Division of Endocrinology and Metabolism, Department of Internal Medicine III, Medical University of Vienna, Vienna, Austria; ^2^Pituitary Center Rotterdam, Endocrinology Section, Department of Internal Medicine, Erasmus University Medical Centre, Rotterdam, Netherlands; ^3^IIB-Sant Pau and Service of Endocrinology, Department of Medicine, Centro de Investigacion Biomedica en Enfermedades Raras (CIBER-ER Unidad 747), Hospital Sant Pau, Universitat Autonoma de Barcelona, Barcelona, Spain; ^4^Neuroendocrine Unit, Massachusetts General Hospital, Boston, MA, United States; ^5^Global Biometrics & Data Management, Pfizer Inc, New York, NY, United States; ^6^Endocrine Care, Pfizer Inc, New York, NY, United States

**Keywords:** hypertension, acromegaly, pegvisomant, prognosis, mortality, cardiovascular disease, hypopituitarism

## Abstract

**Context:**

Hypertension is a major cardiovascular risk factor related to increased mortality in acromegaly. Surgical cure of acromegaly is associated with improvement in blood pressure levels, however little is known about the effect of pegvisomant (PEGV) treatment in patients with hypertension. This analysis evaluates outcomes in patients with hypertension and acromegaly included in ACROSTUDY.

**Methods:**

ACROSTUDY is a global non-interventional surveillance study of long-term treatment with PEGV, monitoring its safety and efficacy. The cohort was retrospectively divided in two subgroups: patients with and without hypertension. Stepwise logistic regression and Kaplan-Meyer analyses were performed for testing predictors of mortality.

**Results:**

The total cohort included 2,090 patients with acromegaly treated with PEGV who were followed for a median of 6.8 years (range up to 12.1 years). In ACROSTUDY there were 1,344 patients with hypertension (52.3% males). This subgroup was older, had a higher BMI, and higher prevalence of diabetes, hyperlipidemia, and cardiovascular disease (CVD) when compared to patients without hypertension. During ACROSTUDY, 68 deaths were reported in the hypertension cohort, vs 10 in the cohort without hypertension. Both CVD (p<0.0001) and anterior pituitary deficiencies (p=0.0105) at study entry independently predicted mortality in patients with acromegaly and hypertension; Kaplan-Meier analysis confirmed that CVD significantly impairs survival.

**Conclusions:**

Hypertension is common in patients with acromegaly and significantly increases mortality, especially when there is concomitant CVD. These data suggest that treatment goals should extend beyond IGF-I normalization, and include optimisation of substitution of pituitary deficiencies and scrutinous screening and treatment of CVD.

## Introduction

Hypertension is a classical cardiovascular risk factor and the most prevalent cardiovascular comorbidity accompanying acromegaly, reported in 15–60% of registry based acromegaly cohorts (1–7). The severity of hypertension in acromegaly is related to GH-IGF-I excess, which is associated with enhanced sodium- and water retention, expansion of plasma volume and increased systemic vascular resistance (8–10).

Both GH hypersecretion and hypertension contribute to the pathophysiology of cardiovascular changes accompanying acromegaly, which include left ventricular hypertrophy, cardiomyopathy, valvular disease, arrhythmias, and heart failure (5, 8). GH hypersecretion is considered the most important causitive factor, as it has a strong impact on heart morphology and function, independent of blood pressure (5, 11, 12). Several studies reported that hypertension plays an additive role aggravating the cardiomyopathy and increasing cardiovascular risk in acromegaly (5, 13). Although cardiac disease is highly prevalent in patients with acromegaly, with over 70% of cases in autopsy studies, and has even been found in asymptomatic patients, several studies have shown that acromegaly per se is not specifically associated with an increased incidence of coronary heart disease (10, 11, 14). Nevertheless, the atherogenic effects of most acromegaly comorbidities such as hypertension, insulin resistance, diabetes, hyperlipidemia and sleep apnea, as well as the acromegaly cardiomyopathy, all contribute to the increased incidence of cardiovascular and cerebrovascular diseases in this population (8, 10, 15).

Acromegaly is also associated with an excess in mortality, predominantly due to cardiovascular and cerebrovascular diseases, and several studies have confirmed that normalisation in GH/IGF-I ameliorates and even nearly normalizes mortality rate (16–18). Mortality in patients with acromegaly is directly associated to IGF-I concentrations, older age, the presence of hypertension and duration of symptoms prior to diagnosis (13, 19). Therefore, the optimal management of acromegaly includes the normalisation of GH/IGF-I levels and disease symptoms, as well as the evaluation and treatment of comorbidities, aiming at reducing morbidity and mortality (20, 21).

Here we prospectively studied patients with acromegaly included in ACROSTUDY™ receiving pegvisomant, aiming at characterising the cohort of patients with hypertension, their biochemical control, comorbidities, and factors related to mortality.

## Patients and Methods

### Study Protocol

ACROSTUDY™ is a global non-interventional surveillance study of long-term treatment with pegvisomant established in 2004 for evaluating outcomes in patients with acromegaly (22, 23). The study prospectively recruited patients in 204 centers from 15 countries and adhered to all applicable local laws and regulatory requirements; local ethical approval was obtained for each participating center and all patients were enrolled after providing written informed consent. Patients were eligible for inclusion if they were on PEGV treatment, or if they were about to start PEGV therapy. Exclusion criteria were participation in any interventional trial and/or tumor mass problems requiring surgical decompression. Study protocol details have been described in previous publications (4, 22, 23). Importantly, decisions on acromegaly treatment were made by the investigators following the routine clinical practice of specific clinics and countries.

Follow-up visits were scheduled following the practice of the treating physician, generally at least once yearly. Case report forms were specially designed for capturing comorbidities and cardiovascular risk factors in addition to acromegaly-specific outcomes, and the following parameters were obtained at baseline (entry in the ACROSTUDY) and during each follow-up visit: blood pressure, weight, height, GH, IGF-I, liver function tests, plasma glucose, HbA1c, comorbidities, current medication (including any changes and reasons for changing), and information on pituitary imaging. At baseline and at each visit, the investigators were asked to confirm the presence or absence of the following cardiovascular risk factors: hypertension, smoking, obesity, diabetes, hyperlipidemia, as well as the presence of cardiovascular and cerebrovascular diseases.

### Mortality

For the present study, we evaluated data from the 2^nd^ interim analysis database freeze on the 12th of May 2016, which included 2090 patients followed up for a median of 6.8 years with a maximum of 12.1 years (23). Data on mortality during the study period were obtained from the adverse report forms, which were evaluated by two independent researchers for verifying the cause of death given by the investigator.

### Hypertension Group

For this analysis, patients were retrospectively divided in two cohorts: Hypertension and Normotension. Patients were included in the Hypertension group if they were identified to have hypertension at any visit during the ACROSTUDY. The presence of hypertension was determined when at least one of the following three was present: 1) hypertension listed as comorbidity by the investigator, 2) antihypertensive medication was used and 3) systolic blood pressure was >140 mm Hg and/or diastolic blood pressure was >90 mm Hg.

### Comorbidities

The presence of comorbidities at ACROSTUDY entry was evaluated by asking the investigators to fill in the study report form with “yes” or “no” for the presence of diabetes, hyperlipidemia, coronary heart disease, coronary angioplasty with or without stent implantation, coronary artery bypass surgery, arrhythmia, heart failure, myocardial infarction, transient ischemic attack, cerebrovascular event, sleep apnea, other respiratory disease, liver disease, gallstones, and cancer. Diabetes was diagnosed if at least one of the following was present: 1) diabetes given as a comorbidity by the investigator, 2) concomitant intake of antidiabetic medication and 3) HbA1c ≥6.5% (48 mmol/mol), or random glucose >200 mg/dl (11.1 mmol/L) as previously described (24). Hyperlipidemia was diagnosed if at least one of the following was present: 1) hyperlipidemia given as comorbidity by the investigator, 2) concomitant intake of lipid-lowering drugs.

Patients were considered to have cardiovascular disease (CVD) at baseline if any of the following was reported at ACROSTUDY entry: left ventricular hypertrophy, cardiomyopathy, aortic valvular disorders, mitral valvular disorders, tricuspid valvular disorders, arrhythmias, myocardial ischaemia, myocardial infarction, heart failure, cerebrovascular accident, transient ischaemic attack, haemorrhagic stroke and ischaemic stroke.

### Statistical Analysis

Continuous measurements were summarized using descriptive statistics and discrete data by counts. Demographic and co-morbidities of acromegaly patients enrolled in the study are summarized descriptively by hypertensive and normotensive groups. As this is a non-interventional study, all tests of significance provided (eg., p-values) are exploratory, not definitive and post-hoc in nature and should be interpreted with caution. Stepwise logistic regression for mortality was performed using independent predictors of demographic, clinical and co-morbid risk factors. Odds ratios and 95% confidence intervals for them are also provided. Kaplan-Meier analyses were performed for the significant predictors of mortality and survival from the stepwise logistic regression analysis for the total cohort of patients and within the hypertensive sub-cohort.

For the purposes of statistical analyses, baseline was defined as the start of PEGV therapy upon entry in the ACROSTUDY. For adverse event (AE) reporting, including serious adverse events (SAEs), death and laboratory results, safety data were assessed at all visits through case report forms and spontaneous reports. Adverse events data were coded using the current MedDRA version available at the time of reporting. All statistical analyses are performed using SAS version 9.4 (25).

## Results

**Figure 1:**
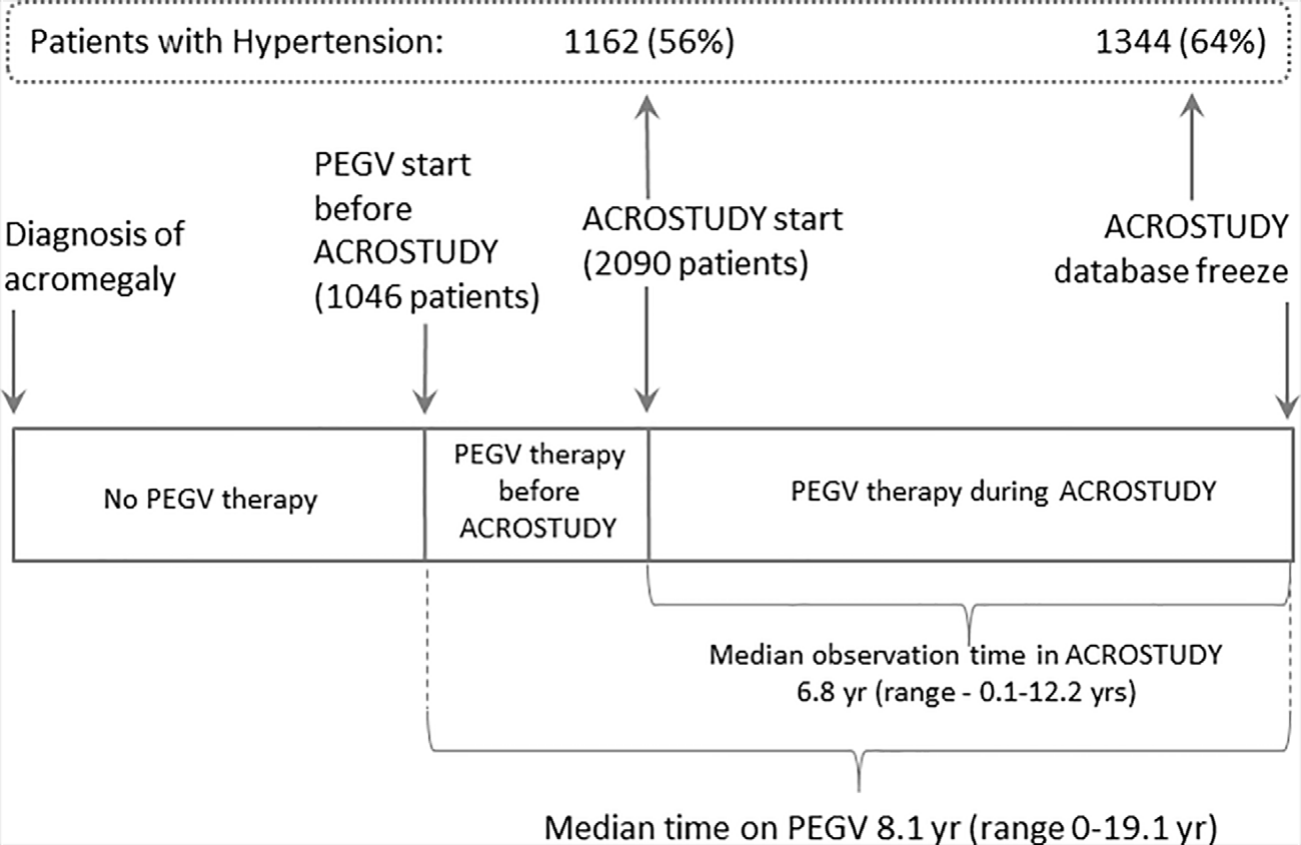
ACROSTUDY flow chart.

Here we analyse data from 2,090 patients followed for a median of 6.8 years (range up to 12.1 yrs), in total, 7,687.5 patient years (**Figure 1**). Data on acromegaly treatment outcomes were already reported previously (23).

### Characteristics of the Hypertension Cohort

The prevalence of hypertension in the ACROSTUDY cohort was 56% at study entry and increased to 64% during the follow-up period, with 182 newly diagnosed patients with hypertension during the study (**Figure 1**). For this analysis, the entire ACROSTUDY population was divided in two groups: 1) patients with hypertension at any time during the study (64% of patients) and 2) patients with normotension at any time during the study (36% of patients). Baseline demographic characteristics of the cohorts with and without hypertension are shown in **Table 1**. At study entry, patients belonging to the hypertension group were older at initial diagnosis of acromegaly, older at study entry, had higher BMI and a higher prevalence of all cardiovascular risk factors (**Table 1**). In addition, patients with hypertension had significantly more pituitary deficiencies and received less pituitary surgery (**Table 1**). Patients who started PEGV ≥5 years after diagnosis had a higher prevalence of hypertension. At baseline and at year 5, the average blood pressure reduced from 137/84 to 134/81 mmHg in Hypertension and 122/76 to 120/75 mmHg in Normotension groups, respectively. 20.4% of patients receiving PEGV monotherapy, 19.7% of patients receiving PEGV + somatostatin receptor ligands (SRL), 20.7% of patients receiving PEGV + dopamine agonists (DA) and 24.1% of patients receiving tripple therapy (PEGV + SRL + DA) developed novel hypertension during the observation time in ACROSTUDY.

**Table 1:** Differences in baseline characteristics, comorbidities and pituitary disease between patients with and without hypertension.

	Hypertensionn=1344	Normotensionn=746	P-value
**Patient characteristics**			
Age at diagnosis of acromegaly (years)	46.0 (13.1)	37.1 (12.4)	<0.0001
Age at PEGV start (yrs)	53.9 (12.9)	44.0 (13.6)	<0.0001
Gender	47.7% ♀	50.7% ♀	0.1723
Ethnicity	92.9% caucasian	93.1% caucasian	0.1428
**CV risk factors and comorbidities**			
Age at ACROSTUDY start (years)	55.6 (12.9)	45.6 (13.7)	<0.0001
BMI (kg/m^2^)	30.5 (5.4)	28.3 (4.9)	<0.0001
Smoking	27.7%	24.9%	<0.0001
Cardiovascular disease	7.4%	2.5%	<0.0001
Diabetes	20.2%	11.4%	<0.0001
Hyperlipidemia	17.0%	5.6%	<0.0001
**Pituitary disease**			
Pituitary surgery	73.7%	82.7%	<0.0001
Radiotherapy	30.9%	30.8%	0.9606
Pituitary deficiencies	53%	47%	0.0224
ACTH deficiency	24%	23%	0.7078
Baseline IGF-I (µg/L)	364 (283)	387 (278)	0.3265
IGF-I > ULN	88.9%	87.4%	0.3924
IGF-I (foldULN = IGF-I/ULN)	1.54 (1.11)	1.39 (0.87)	0.0841

Data are shown as mean (SD) or percentage. CV, cardiovascular; CVD, cardiovascular disease; IGF-I, insulin-like growth factor-I; PEGV, pegvisomant; ULN, upper limit of normal.

### Acromegaly Control Rates in the Hypertension Cohort

**Figure 2:**
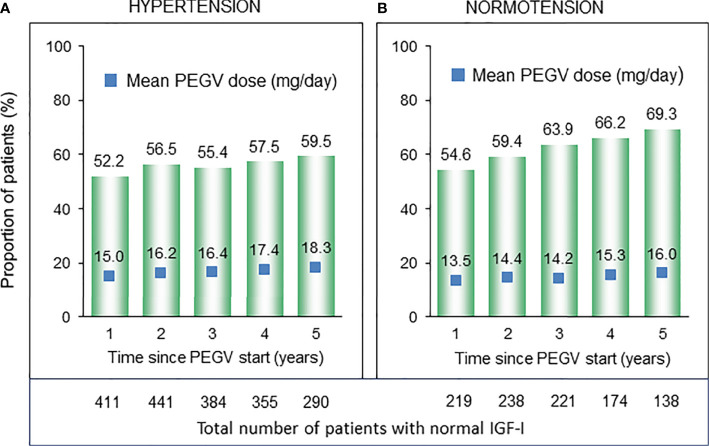
Number and proportion of patients with normal IGF-I (bar charts) and respective PEGV (pegvisomant) dose (blue boxes) in the subcohorts of patients with **(A)** and without **(B)** hypertension.

At entry into the ACROSTUDY, IGF-I values evaluated as fold-ULN were similar in both groups (**Table 1**). During the follow-up period, the percentages of patients with IGF-I within the normal range continously increased in both groups, changing from 52.2 to 59.5% in the Hypertension group and from 54.6 to 69.3% in the Normotension group, but were significantly higher in the Normotension group when compared to the Hypertension group 1–5 years after starting PEGV (p<0.001 (**Figure 2**). Hypertensive patients received significantly higher PEGV doses (p=0.014) for the first five years after the start of Somavert therapy ranging from 15 to 18.3 mg/day, when compared to 13.5 to 16 mg/day in the Normotension group.

### Mortality in the Hypertension Cohort

All adverse effects observed in ACROSTUDY are shown in **Table 2**. They were also previously reported for the overall population by Buchfelder et al. (23). A total of 78 deaths (56% in males) were observed: 68 in the Hypertension group (5.1%) and 10 in the Normotension group (1.3%), and mortality rate was 13.2 per 1,000 patient years in the Hypertension group and 4 per 1,000 patient years in the Normotension group. The causes of death were predominantly cardiovascular (31%), cerebrovascular (18%), cancer (18%) and respiratory problems/sepsis (13%). For further analysis, both cardiovascular and cerebrovascular causes of death are classified as cardiovascular deaths, and included cardiac arrest, ventricular fibrillation, myocardial infarction, heart failure, aortic aneurysm rupture, cardiogenic shock, bilateral massive pulmonary embolism, stroke/cerebrovascular accident, basilar vein thrombosis and subarachnoidal haemorrhage. The most common cause of death due to CVD was heart failure, which accounted for 9 cases. Age of death ranged from 35.6 to 88.6 years (mean 68.4 years, SD 12.4). Two deaths occurred before the age of 40 and were caused by ventricular fibrillation in one patient and aortic aneurysm rupture in the other. Female patients died at an older age, with a mean age at death of 71.8 years (SD 13.2) for females and 65.9 years (SD 11.3) for males. No gender differences were observed in the distribution of causes of death.

**Table 2:** Adverse events in patients with and without hypertension.

	HypertensionN (%)	NormotensionN (%)
**Adverse effects (AEs)**		
Subjects with AEs (all cause)	773 (57.5%)	364 (48.8%)
Subjects with treatment-related AEs	206 (15.3%)	131 (17.6%)
**Serious adverse effects (SAEs)**		
Subjects with SAEs (all cause)	343 (25.5%)	116 (15.5%)
Subjects with treatment-related SAEs	35 (2.6%)	14 (1.9%)
Deaths (all non-treatment related)	68 (5.1%)	10 (1.3%)
**Drugs withdrawn due to SAEs**		
Discontinued due to SAEs	116 (8.6%)	30 (4%)
Discontinued due to treatment-related SAEs	19 (1.4%)	5 (0.7%)

Data are shown as total numbers and respective percentages. Treatment=Pegvisomant.

For identifying which factors influenced the increased mortality in the hypertension cohort we performed a univariate analysis testing the relationship between mortality and baseline factors obtained at the ACROSTUDY entry visit. Older age, the concomitant presence of pituitary deficiencies, diabetes, hyperlipidemia, and CVD were all significantly related to mortality (**Table 3**). Gender, BMI, smoking habits and IGF-I levels were not associated with mortality.

**Table 3:** Factors related to mortality in hypertensive patients with acromegaly.

	Odds Ratio	95% confidence limits for Odds Ratio		P-value
**Univariate analysis**				
Age at diagnosis of acromegaly (years)	1.008	0.982	1.034	<0.0001
Age at ACROSTUDY start (yrs)	0.868	0.767	0.982	0.0247
Gender	0.797	0.487	1.304	0.3662
BMI	0.972	0.929	1.016	0.2105
Smoking	0.963	0.568	1.633	0.8895
Diabetes	0.558	0.342	0.910	0.0194
Hyperlipidemia	3.263	1.014	10.500	0.0474
Cardiovascular disease	0.124	0.071	0.215	<0.0001
IGF-I at study entry (µg/L)	1.001	0.999	1.002	0.3252
IGF-I at last visit (µg/L)	0.999	0.997	1.001	0.1997
Pituitary deficiencies	0.415	0.242	0.714	0.0015
ACTH deficiency	0.652	0.401	1.060	0.0844
**Step-wise logistic regression analysis**				
Age at diagnosis of acromegaly (years)	0.931	0.906	0.958	<0.0001
Cardiovascular disease	0.145	0.068	0.308	<0.0001
Pituitary deficiencies	0.378	0.179	0.796	0.0105

Using step-wise logistic regression analyses with all-cause-mortality as the outcome measure, we found that only age at diagnosis of acromegaly, the presence of CVD and pituitary deficiencies independently predicted mortality (**Table 3**). In the Hypertension cohort, 34% of patients who died had already been diagnosed with CVD at study entry. In the cohort of hypertensive patients who survived, CVD was present in less then 6%. Pituitary deficiency was present in 72% of hypertensive patients who died, and in 51% of survivors. Traditional cardiovascular risk factors such as smoking, BMI and gender were not associated to mortality in acromegaly patients with hypertension.

**Figure 3:**
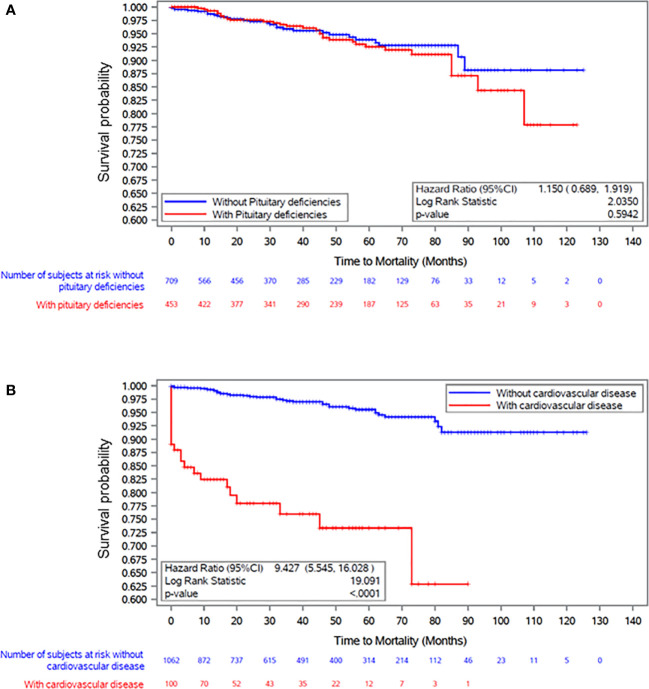
Kaplan Meier plot survival curves in patients with acromegaly and hypertension in relation to the presence of **(A)** pituitary deficiencies and **(B)** cardiovascular disease at baseline visit (ACROSTUDY entry).

To further explore the importance of CVD and pituitary deficiencies on mortality, we performed a Kaplan-Meyer analysis testing the differences in survival curves between hypertensive patients with and without pituitary deficiencies and CVD (**Figure 3**). Survival in patients with acromegaly and hypertension was not significantly influenced by concomitant hypopituitarism (**Figure 3A**). The presence of CVD led to a remarkable reduction in survival (**Figure 3B**).

## Discussion

Previous studies have identified hypertension as an independent factor related to an increased mortality in patients with acromegaly (13, 26). Here we report the data from a large international cohort study of patients with a long history of acromegaly, necessitating long-term acromegaly-specific therapies. We observed a high prevalence of hypertension, which increases during the follow-up period, as patients get older. In this cohort, the presence of hypertension is associated with ageing and with a higher prevalence of diabetes, hyperlipidemia and other cardiovascular risk factors. After five years, blood pressure values decreased in the whole ACROSTUDY cohort. In the subgroup of patients with acromegaly and hypertension, the presence of CVD was the most robust independent predictor of mortality in both logistic regression and Kaplan-Meier analysis.

Improvements in glucose metabolism and blood pressure observed after successful treatment interventions of acromegaly with PEGV corroborate a direct link between GH-IGF-I excess and hypertension and diabetes (6, 15, 24, 27, 28). Indeed, long-term excess in GH/IGF-I plays an important role in the development of hypertension, insulin resistance, diabetes and obesity, which in turn also impact each other (8, 10, 15). As PEGV is commonly used as a second- or third-line therapy in acromegaly, most patients included in ACROSTUDY were either not adequately controlled with other treatment modalities or could not receive/tolerate other treatments. This may explain why ACROSTUDY patients were older and had active disease longer than other cohorts studied to date; this may also be the reason that the ACROSTUDY population had a higher prevalence of acromegaly-associated comorbidities at baseline when compared to other cohorts (4, 22, 23). The long follow-up period in the study extending to up to 12.2 years allowed us to identify a large number of patients with hypertension, which reached 1,344 patients (64% of the cohort) at the time of the study data freeze for the second interim analysis. The high prevalence of arterial hypertension in the ACROSTUDY cohort, which has a high frequency of IGF-I normalization, and its relationship to age, higher BMI, smoking and other comorbidities, corroborates the multifactorial pathophysiology of hypertension in patients with acromegaly, comparable to the general population. To our knowledge, this is the largest cohort of patients with acromegaly and hypertension studied to date.

Based on already published reports and our clinical experience, hypertension in acromegaly is usually not very severe, and can be effectively controlled with available antihypertensive medications (6). This is also confirmed by our observations on a small decrease in blood pressure values during ACROSTUDY. Nevertheless, hypertension plays an important pathophysiological role in the development of acromegaly-associated comorbidities, such as insulin resistance, diabetes and CVD (6, 8). Indeed, in the present study we found significantly higher PEGV doses in patients with hypertension. The main reasons for this might be the larger prevalence of diabetes and the higher BMI in patients with hypertension and acromegaly, when compared to patients without hypertension, as the presence of diabetes necessitates higher PEGV doses for achieving biochemical control (29). In terms of pituitary disease, the most significant difference was the fact that patients with hypertension had undergone significantly less pituitary surgery. Although the reason for this was not captured, it may be related to the older age and multiple comorbidities found in the hypertensive population and is in agreement with previous reports on acromegaly in older patients (30).

At entry in ACROSTUDY the prevalence of CVD reported in the sub-cohort of patients with acromegaly and hypertension was 7.4%. Cardiac disease in acromegaly was found by other investigators to depend on the amount and extent of GH hypersecretion, and was often partly reversible after normalization of GH levels *via* surgery or by blocking GH action *via* PEGV (31). Hypertension plays an additive role, aggravating GH/IGF-I-induced heart disease (5). A previous study specifically analyzing all acromegaly patients for the presence of CVD reported that all patients with hypertension and diabetes had echocardiographic abnormalities (8). In clinical practice, testing for the presence of CVD necessitates referrals to other specialists and is not systematically done by the internist/endocrinologist. Patients with acromegaly were found to have morphological cardiac disease in the absence of signs and symptoms. So we cannot exclude that clinically silent but functional or morphological CVD might be underdiagnosed in our population. Our data show that the prevalence of reported CVD in the subcohort of patients with acromegaly and normotension is not higher than in the general normotensive population, while the prevalence of reported CVD in patients with acromegaly and hypertension is similar to the general population with well-treated hypertension (32). This evidence supports the fact that arterial hypertension appears to be the main factor contributing to the development of CVD in this cohort of patients with acromegaly, who have a high frequency of IGF-I normalization. In addition, our data are in line with recent studies finding a lower frequency of CVD in acromegaly than described in older cohorts, mainly attributed to better disease control due to novel treatment modalities (10).

The current guidelines for diagnosing and treating acromegaly promote screening all patients for the presence of comorbidities, including CVD (20). Mortality in acromegaly has improved during the last decades and is considered to be similar to the normal population in well-controlled patients, and this is linked to the lower prevalence of CVD (10, 18, 33). Here we show that the presence of hypertension is associated with a 3.3 fold increased mortality rate in patients with acromegaly, nevertheless mortality rate in the cohort of patients with acromegaly and hypertension appears similar to the mortality rate of the general population with hypertension (34).

The strength of the present study is the prospective design for capturing acromegaly comorbidities, and the inclusion of a large number of patients with uncontrolled acromegaly, which led to a high number of comorbidities and mortality events. Additional strengths are the long-term follow up, the real-world setting with inclusion of patients that may not have been enrolled in clinical trials. Limitations include the retrospective classification of Hypertension or Normotension groups, the investigator-based definition of cardiovascular disease, the lack of information on how adequately pituitary deficiencies were treated and the fact that all patients were on PEGV, so there was no treatment comparator group. Additional strengths/limitations of ACROSTUDY overall were described in the previous publication (23).

Taken together, here we report data on a very large cohort of patients with acromegaly and hypertension, describing a higher prevalence of other comorbidities and cardiovascular risk factors and a 3.3 fold increased mortality when compared to patients with acromegaly without hypertension. Our findings indicate that hypertension significantly increases mortality in patients with acromegaly, especially in the presence of concomitant CVD, highlighting the importance of testing for and treating cardiac disease in hypertensive patients with acromegaly.

## Data Availability Statement

Upon request, and subject to certain criteria, conditions, and exceptions (see https://www.pfizer.com/science/clinical-trials/trial-data-and-results for more information), Pfizer will provide access to individual de-identified participant data from Pfizer-sponsored global interventional clinical studies conducted for medicines, vaccines, and medical devices (1.) for indications that have been approved in the US and/or EU or (2.) in programs that have been terminated (i.e. development for all indications has been discontinued). Pfizer will also consider requests for the protocol, data dictionary, and statistical analysis plan. Data may be requested from Pfizer trials 24 months after study completion. The de-identified participant data will be made available to researchers whose proposals meet the research criteria and other conditions, and for which an exception does not apply, via a secure portal. To gain access, data requestors must enter into a data access agreement with Pfizer.

## Ethics Statement

The studies involving human participants were reviewed and approved by Ethics Committee of the Medical University of Vienna and local Ethics Committees in each participating center. The patients/participants provided their written informed consent to participate in this study.

## Author Contributions

JH-H and GV conceived this study, which was approved by the ACROSTUDY steering committee. All authors critically revised the study protocol. SV, JH-H, and GV collected and analyzed the data. GV wrote the initial version of the manuscript. All authors contributed to the article and approved the submitted version.

## Funding

ACROSTUDY was sponsored by Pfizer.

## Conflict of Interest

GV has received research grants to her institution from Chiasma and Novartis, and consultancy- and/or speakers fees from Pfizer, Novartis and Ipsen. AL has received lecture and/or consulting honoraria from Ipsen, Novartis, and Pfizer; research grants to the Medical University of Vienna from Ipsen and Pfizer. AJL has received grants, consultancy- and speaker’s fees from Pfizer. SN has received research grants and speaker fees from Ipsen, Novartis, and Pfizer, and consulting fees from Ipsen. SW has received honoraria as a member of the Acrostudy Advisory Board, and lecture fees from Pfizer, Novartis and Ipsen. BB has served as PI of research grants from Novartis, Crinetics and Ionis to Massachusetts General Hospital and has received occasional consulting honoraria from Novartis, Crinetics and Pfizer. SV and JH-H are Pfizer employees.
